# “Could Patient Age and Gender, along with Mass Size, Be Predictive Factors for Benign Kidney Tumors?”: A Retrospective Analysis of 307 Consecutive Single Renal Masses Treated with Partial or Radical Nephrectomy

**DOI:** 10.3390/bioengineering10070794

**Published:** 2023-07-03

**Authors:** Raffaele Baio, Giovanni Molisso, Christian Caruana, Umberto Di Mauro, Olivier Intilla, Umberto Pane, Costantino D’Angelo, Antonio Campitelli, Francesca Pentimalli, Roberto Sanseverino

**Affiliations:** 1 Department of Medicine and Surgery “Scuola Medica Salernitana”, University of Salerno, 84081 Salerno, Italy; 2 Department of Urology, Umberto I, Nocera Inferiore, 84014 Salerno, Italy; 3 Department of Chemistry, University of Malta, MSD2080 Msida, Malta; 4 Department of Medical Biotechnologies, University of Siena, 53100 Siena, Italy; 5 Department of Medicine and Surgery, LUM University, 70010 Bari, Italy

**Keywords:** laparoscopic partial nephrectomy, benign renal mass, tumor size

## Abstract

Due to the increased use of common and non-invasive abdominal imaging techniques over the last few decades, the diagnosis of about 60% of renal tumors is incidental. Contrast-enhancing renal nodules on computed tomography are diagnosed as malignant tumors, which are often removed surgically without first performing a biopsy. Most kidney nodules are renal cell carcinoma (RCC) after surgical treatment, but a non-negligible rate of these nodules may be benign on final pathology; as a result, patients undergo unnecessary surgery with an associated significant morbidity. Our study aimed to identify a subgroup of patients with higher odds of harboring benign tumors, who would hence benefit from further diagnostic examinations (such as renal biopsy) or active surveillance. We performed a retrospective review of the medical data, including pathology results, of patients undergoing surgery for solid renal masses that were suspected to be RCCs (for a total sample of 307 patients). Owing to the widespread use of common and non-invasive imaging techniques, the incidental diagnosis of kidney tumors has become increasingly common. Considering that a non-negligible rate of these tumors is found to be benign after surgery at pathological examination, it is crucial to identify features that can correctly diagnose a mass as benign or not. According to our study results, female sex and tumor size ≤ 3 cm were independent predictors of benign disease. Contrary to that demonstrated by other authors, increasing patient age was also positively linked to a greater risk of malign pathology.

## 1. Introduction

The exponential increase in the use of common and non-invasive abdominal imaging techniques (computed tomography [CT], ultrasonography and magnetic resonance imaging [MRI]) over the last few decades has led to a similar increase in the number of small, often asymptomatic, renal tumors incidentally detected that generally migrate to the lower clinical stage [1,2]. All of this results in a risk of cancer overdiagnosis. In addition, according to studies from Western countries, 15–20% of small kidney tumors show benign nature on definitive pathological examination [3,4,5] and frequently, also when they have malign nature, these small renal masses (SRMs) present an indolent course [6]. To date, it is not possible to uniquely diagnose a tumor as malignant or benign by specific imaging findings [7,8]. Commonly, a CT scan is needed in the assessment of kidney nodules [9,10]; at this radiological examination, contrast-enhancing renal nodules are classified as malignant and they are often removed surgically without first performing a biopsy. In an effort to improve diagnostic accuracy, several methods in the radiological field have been evaluated [11]; considering that, according to Remzi et al. [12], only 17% of kidney nodules are correctly classified as benign on pre-operative CT (resulting in a subsequent overtreatment with radical nephrectomy in the 43% of these nodules). This is because most kidney nodules are renal cell carcinoma (RCC) after surgical treatment, but a non-negligible rate of these nodules may show benign nature on final pathology; this concern takes on greater significance if we consider the not uncommon probability of benign nature even in the case of kidney nodules that have a diameter of >4 cm. As a result, some patients undergo unnecessary surgery with associated significant morbidity. This has an important impact on health policy [13]. This scenario has prompted a choice of partial nephrectomy (PN) for the surgical treatment of these masses. Consequently, since 2009, both American and European guidelines have recommended PN as the gold standard for patients with T1 masses that can be feasibly excised [14], and since 2014, this indication has also been extended to T2 lesions that are technically susceptible to PN. In effect, compared with radical surgery, PN guarantees similar oncological outcomes, especially for those patients suffering from T1 renal cell carcinoma (RCC) [15,16,17,18]. The choice of PN makes it possible to reduce the risk of performing radical surgery (with the consequent removal of the kidney) in the case of benign neoplasia, although the latter may have larger diameters. With respect to this, in our previous research [19], the medical records of patients undergoing laparoscopic PN at our department over the last 10 years were retrospectively reviewed, including a total of 195 patients. The analysis result was that 30 (15.4%) of the 195 kidney lesions removed were diagnosed as benign by the pathologist, with a complication rate of 10% (3/30 cases) among these patients. In effect, in one case, conversion to open surgery was required due to uncontrollable bleeding while, in two other patients, the placement of a double-J pyelo-ureteral stent was needed for the urinary fistula during the post-operative time. This last data about the complication rate represents another interesting and not negligible point of reflection. According to the results of a review of nine studies including >1000 patients, PN was associated with low but fundamentally operative morbidity and mortality rates [8]. The types of complications, in decreasing order of incidence rate, based on this review [8], are urinary fistula, infection or abscess, post-operative bleeding, reintervention and peri-operative death. Another study assessed 180 patients undergoing PN [20]: after surgery, hemorrhage was reported in 4 (2.2%) patients while urinary fistula in other 3 (1.7%) patients. Furthermore, as demonstrated by our previous research [21], in addition to the complications listed above, during renal surgery, even if it is performed with a laparoscopic approach, an infrequent complication such as pneumothorax is a potential risk. In fact, in our retrospective study including 384 laparoscopic nephrectomies, in a total of four patients (1.04%) diaphragmatic injury was found (requiring intracorporeal suturing). For all these reasons, if on the one hand, the radical nephrectomy is not to be considered a choice of treatment for small kidney nodules that can be easily excised; on the other hand, PN is a valid tool for both the diagnosis and treatment of these small renal masses, but its suitability for all suspicious masses remains questionable due to the significant impact of benign tumors and the morbidity rate associated with this type of surgery. In conclusion, for patients suffering from suspected renal lesions, the treatment planning is uncertain due to the failure of current imaging techniques and renal biopsy to accurately distinguish RCCs from benign tumors before surgery [1,22]. Surveillance might be an appropriate alternative to immediate surgery for patients with indolent tumors, considering the low risk of progression if the treatment is delayed [6]. In fact, patients under surveillance rarely progress to metastatic disease [23]. However, the choice of which patients are suitable for surveillance can be difficult because the distinction between benign masses or indolent malignant tumors and aggressive cancer may not be possible without complete surgical removal. For evaluating the potential aggressiveness of small renal masses, several tools have been used to facilitate the choice between surveillance and immediate surgery. The current retrospective report describes the incidence and predictive factors of benign renal masses by surgery (performed with both laparoscopic and open approaches). Understanding this analysis allows us to identify which patients are more likely to have benign tumors and who would therefore benefit from additional diagnostic methods (such as renal biopsy) or active surveillance.

## 2. Study Sample

This retrospective study was conducted at the Department of Urology of Umberto I Hospital; written informed consent was obtained from each patient. The study sample was composed of patients who underwent surgery for the removal of a suspected renal cell carcinoma. Overall, 307 patients at our department met the inclusion criteria for enrollment in the study. The patients underwent radical or partial nephrectomy (either using the laparoscopic approach for almost all cases or the open approach in the very few patients who were not candidates for various problems) due to the pre-operative suspicion of RCC, according to the radiographic aspect of the renal mass on ultrasonography and CT scan. All operations (laparoscopic or open) were performed using a transperitoneal or retroperitoneal approach based on the patient’s history of abdominal surgery, habitus, tumor location and surgeon preference. Furthermore, in the case of partial nephrectomy, for better preserving the kidney function, the off-clamp technique was chosen in all cases (with patients under controlled hypotension). If a pre-operative diagnosis could not be made with the above imaging techniques, magnetic resonance (MR) was performed to provide further details. Three criteria were used to raise suspicion of renal cell carcinoma for surgical treatment: (1) solid enhanced nodules (showing an increase of 10 Hounsfield units at CT scan); (2) absence of intratumor fat in the kidney lesions in order to exclude angiomyolipomas and (3) complicated renal cysts of type III or IV (Bosniak classification [24]). Exclusion criteria were genetic predisposition for von Hippel–Lindau disease or Birt–Hogg–Dube syndrome, tumor biopsy, primary urothelial cell carcinoma, metastatic tumors, tuberous sclerosis or patients with multiple or bilateral nodules, kidney cysts or if the solid kidney nodule was classified as angiomyolipoma by evidence of fat on the pre-surgery CT scan. Our analysis included patients’ demographic data (age and gender) and the following tumor features: size of mass (widest diameter), body site affected (right or left kidney), histology and year of surgery.

## 3. Materials and Methods

Treatment-independent patient characteristics considered in this study were sex, age and year of treatment. Variables related to the treatment were intervention type, removal type and kidney location (left vs. right), while the renal tumor was characterized by its histology and size. The primary goal of the study was the histological characterization of the tumor into benign or malign (on which a different method of medical management of the patient depends), and all analyses were directed towards understanding its relationship with the other variables with the aim of identifying subgroups in the data which present reduced malignancy risk. The statistical analysis was divided into two main steps. First, numbers and proportions were reported for categorical variables, while continuous variables were described by their mean and standard deviation. This was followed by an exploration of the correlation between the various variables via a correlation map that was constructed using Spearman’s correlation test. In the second part of the analysis, both univariate and multivariate logistic regression were used to evaluate the impact of each variable on the probability of malignancy. In both cases, age and tumor size were considered both as continuous variables and as categorized variables. All statistical analyses were carried out using the RStudio graphical interface version 2021.09.2 Build 328 with R version 3.5.2 (R Foundation, Vienna, Austria).

## 4. Results

**Figure 1:**
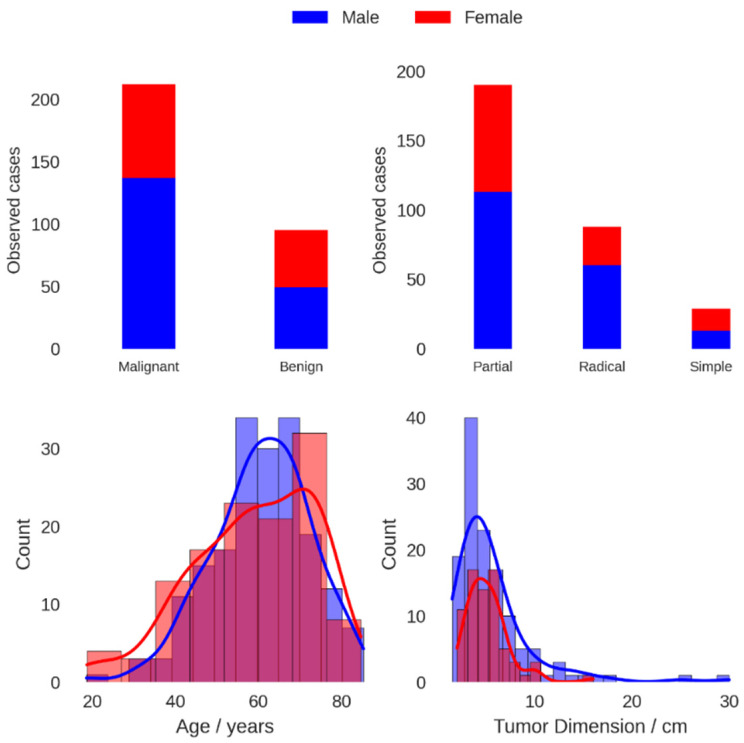
Tumor malignancy (**upper left**), intervention type (**upper right**), histogram and kernel density estimates for age (**lower left**) and tumor size (**lower right**) for male (blue) and female (red) patients.

**Figure 2:**
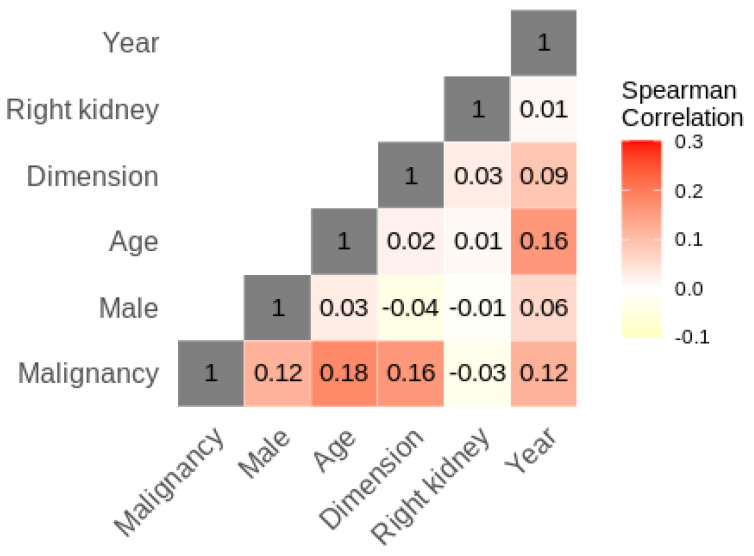
Patient characteristic Spearman correlation heatmap.

Males represented the majority of the patients at 60.6%. The overall mean age (SD) was 60 (13) years. The number of malignant cases was 212, which represented 69% of the cases overall. The majority of malign cases were attributed to RCC at 72.6%, with papillary tumors at 12.3%, chromophobe tumors at 7.5% and other types of malign tumors comprising 7.5% of malign cases. For the benign conditions, 35.8% were oncocytomas, 22.1% were pyelonephritis, 11.6% were angiomyolipomas, 10.5% were hydronephrosis and 20% were other types of benign conditions. Table 1 provides a summary of these findings, while distribution characteristics for select parameters are shown graphically in Figure 1. Logistic modeling results are given in Table 2 for univariate regression and Table 3 for multivariate regression. Univariate logistic regression showed that increasing tumor size, male sex and increasing age were all positively linked to an increase in malignancy risk. In particular, when categorized, lesions > 5 cm were the only category with a statistically significant (*p* = 0.019) coefficient difference when compared to the reference group of ≤3 cm, while age < 50 years was the only age category with a statistically significant coefficient difference when compared to the reference group of ≥70 years. In univariate analysis, the year of intervention was also positively associated with an increase in malignancy risk. The results are reported uncorrected for multiple testing. Correlation analysis, presented in Figure 2, showed that the year of analysis was positively correlated with patient age, tumor dimension and male sex, all of which inferred an increased malignancy risk. The univariate result for the year of intervention may thus be explained in terms of the demographic changes occurring in the sample over time. In multivariate analysis, both patient age and cancer dimension were positively linked to increased malignancy risk when treated as continuous variables, while only tumor size > 5 cm remained statistically significant when the variables were categorized. Modeled risk and risk ratios for simulated patients with median characteristics differing in sex, age and/or tumor size, based on the continuous multivariate logistic model, are given in Table 4. A 61.2-year-old female with a 4.6 cm tumor was used as a reference. Compared to this reference, a male presenting the same characteristics has a risk ratio of 1.15, while a 69.75-year-old female has a risk ratio of 1.07. Increasing the tumor size to 6 cm (and maintaining age at 61.2 years) gives a risk ratio of 1.08 for females and 1.21 for males.

## 5. Discussion

In the past, according to urologists, >90% of solid renal masses were RCC at surgery. However, daily results show that, after surgical treatment, up to 27% of suspected kidney nodules are diagnosed as benign tumors by pathologists, and this scenario must be carefully evaluated if we consider that, due to the widespread use of common and non-invasive imaging techniques, the incidental diagnosis of kidney mass has become increasingly common. So, for this reason, it is crucial to identify features that can correctly diagnose a mass as benign or not. In our study, the patient’s age and tumor size were considered both as continuous variables and categorized variables, which allowed for a more comprehensive understanding of the factors influencing the likelihood of malignancy. The use of logistic regression allowed for the identification of variables that were strongly associated with malignancy, which could be used to develop strategies for assessing risk in different subgroups. It is commonly believed that the problem of diagnosis can be solved with the use of renal biopsy. However, Patel et al. [25] reported that about 80% of patients did not undergo surgery after the benign biopsy result; however, following PN, 36.7% of patients with negative biopsy results suffered from malignant disease on surgical specimens. To overcome this problem, based on the results from some authors, chemical differences (detected in human kidney biopsies using two-dimensional correlated spectroscopy) allow to distinguish between aggressive and indolent tumor subtypes in masses localized to the kidney [11]. In addition to improvements in radiology, we believe that clinical and anatomical features (such as the patient’s sex and age, tumor size and location) can be used as predictive factors of benign disease in patients suffering from solitary solid kidney lesions, helping the urologists to better select in which cases the renal biopsy is useful. In this way, we strongly believe that the rate of negative biopsy results, despite the presence of malignant kidney disease, could be decreased. The need to identify factors of any nature capable of predicting whether or not a suspected lesion is malignant or capable of predicting the different degrees of malignancy (low, moderate or high) is common to all medical branches and all neoplastic pathologies. According to the results of the present report, we have found that female sex and tumor size ≤ 3 cm are independent factors capable of predicting the benign nature of a renal mass. Contrary to that demonstrated by P. Violette et al. [26], increasing patients’ age was also positively associated with a greater risk of renal malign pathology. Overall, our results were in agreement with the previous series. In effect, according to Zisman et al. [27], female sex was linked to an increased probability of non-malignant tumors, as in another study in which women had a 27.3% probability of being affected by a benign tumor, while in men the probability was 14.5% [28]. A Japanese study evaluated patients undergoing PN for small kidney nodules: unlike 5.4% of men, 26.1% of women were affected by benign tumors [29]. According to the literature, female gender is considered uniformly protective against renal malignant tumors; however, the impact of mass size on the probability of malignant nature is less codified. In our study, tumor size ≤ 3 cm was linked to a decreased risk of malignant nature, with smaller kidney masses being more likely to be benign. This result was in agreement with other series [30,31], with a significant correlation between benign pathology and tumor size [30]. Furthermore, by increasing the tumor diameter with every centimeter, the probability of renal cell carcinoma (as opposed to a benign tumor) increased by 17% [31]. However, other series have failed to demonstrate this correlation [4,27,28,32]. According to the findings of our report, women with renal masses ≤ 3 cm will likely have a benign tumor on pathological examination. Consequently, the risk of such women being exposed to surgery and its potential morbidity unnecessarily is significant. In order to avoid this risk, further investigations may be useful; an example of this is renal biopsy, considering its improved accuracy and safety and low false-negative rates [33,34]. However, although the performance of renal biopsy to predict RCC subtypes is excellent, with a success rate of 90% in some studies [35], the same results have not been obtained for tumor grade evaluation and, therefore, biological risk [36,37,38,39,40]. Indeed, unfortunately, the diagnosis of low-grade malignant tumors on renal biopsy does not yet exclude high-grade lesions [35]. In the case of small tumors, rather than surgery directly, surveillance may be chosen as an alternative approach. Indeed, according to some authors, selected patients affected by small kidney nodules can be managed in this way in absolute safety [41,42], as also demonstrated by Chawla et al. [23] in their assessment of the behavior of small kidney nodules, from which only three cases of metastases were reported. In addition, in a prospective study, Jewett et al. [43], with biopsies and radiological examinations performed over time, evaluated the rate of progression and metastases in patients affected by small kidney nodules in an overall study sample of 127 patients. The results showed that only 25 patients showed local disease progression while two developed metastatic disease. The above studies further support the hypothesis that small kidney tumors have a slow growth, with a low probability of developing metastases. Consequently, adding the results of the present study to data from the existing literature, active surveillance may be even more highly recommended for older women affected by small kidney nodules (especially those ≤ 3 cm) due to their high odds of harboring benign or clinically indolent tumors. Another alternative option of treatment for patients suffering from suspected renal cancer is represented by percutaneous surgeries: cryoablation and radiofrequency ablation. Even though metastasis and not noticing RCC have occurred in a relatively small rate of patients undergoing this type of surgical approach [44,45], the occurrence of regional relapse following the percutaneous surgeries has been shown to be higher compared with that following nephrectomy (both partial and radical). These data highlight the relevance of the careful employment of these surgical treatments [45]. However, according to Andrea Piasentin et al. [46], percutaneous cryoablation (PCA) can cause killing tumoral cells together with preserving renal function and reducing procedural-related morbidity (the so-called “Trifecta”). In this multi-institutional series, they found that PCA is more likely to achieve good outcomes measured by Trifecta in masses < 2.5 cm than in the bigger one. A major limitation was represented by the high rate of recurrences. In light of these results, in patients not suitable for PN and affected by kidney nodules < 3 cm, PCA can be a valid surgical treatment. Furthermore, stereotactic body radiation therapy (SBRT) is increasingly utilized in the management of localized kidney cancers, particularly for patients who are not surgical candidates. A narrative review of SBRT [47] underlines the safety and efficacy of it in the management of renal tumors (a disease previously thought to be radioresistant). According to the studies included in the review, SBRT can be utilized successfully in the treatment of large renal tumors (>5 cm) and local control is greater than 90%, with rare grade 3 or 4 toxicity and no grade 5 toxicity. As in other reports, we found a correlation between young age and the probability of benign or less aggressive tumors. All of these findings indicate that these patients should be considered suitable candidates for a pre-operative biopsy of the renal mass or active surveillance. This medical strategy would also help in making decisions about future treatment options [48,49,50]. Future prospective studies should therefore evaluate whether a pre-operative renal biopsy or active surveillance in patients at high risk of benign pathological findings reduces the incidence of indications for surgery. The strengths of the present study include its large sample size and the analysis of baseline patient and tumor characteristics, which have an important clinical impact. The limit of our study is its retrospective nature, with intrinsically associated biases. However, we used radiological specimen dimensions (at TC scan) to quantify tumor size. This probably allowed us not to underestimate the effective size of the masses in vivo, because formalin fixation causes tumors to shrink. This is noteworthy because previous studies, which examined non-small-cell lung cancer, have shown the ability of formalin to cause sufficient shrinkage to determine stage migration in some tumors [51]. Lastly, from the univariate logistic regression analysis, it emerged that there was a statistically significant increase in malignancy with an increase in the year of surgery. It should be noted, however, that the correlation heatmap in Figure 2 shows that the year of surgery is positively correlated with age, male sex and tumor size (three factors that have been identified as increasing the risk of malignancy). The increase in malignancy with treatment year can therefore be explained by the sample being progressively composed of older male patients with larger masses at imaging; another explanation for this increase in malignancy could consist in the higher ability of imaging methods to identify a renal nodule and classify it as malign (owing to the greater experience of radiologists and the most advanced instruments).

## 6. Conclusions

In conclusion, the incidental diagnosis of small renal tumors represents a challenge to urologists due to the likelihood that many of these tumors are benign or show less aggressive behavior. We identified a subgroup of patients, namely young women affected by tumors ≤ 3 cm, with a significantly reduced probability of harboring malignant tumors. Thus, according to our findings, significant predictive factors for benign kidney pathology include younger age (<50 years), female gender and small tumor size (≤3 cm), and the choice of renal biopsy and active surveillance may therefore be more strongly recommended for the patients with these characteristics.

## Figures and Tables

**Table 1:** Patient demographic data.

Population Characteristics	
Gender, *n* (%)	
Female	121 (39.4)
Male	186 (60.6)
Age, mean (SD), years	60 (13)
Histological type: malignant tumors, *n* (%)	
RCC	154 (72.6)
Papillary	26 (12.3)
Chromophobe	16 (7.5)
Others	16 (7.5)
Histological type: benign conditions, *n* (%)	
Oncocytoma	34 (35.8)
Pyelonephritis	21 (22.1)
Angiomyolipoma	11 (11.6)
Hydronephrosis	10 (10.5)
Others	19 (20.0)
Intervention type: malignant tumors, *n* (%)	
Laparo	189 (89.2)
Open	20 (9.4)
Removal type: malignant tumors, *n* (%)	
Partial	135 (63.7)
Radical	76 (35.8)
Simple	1 (0.5)
Tumor size malignant, mean (SD), cm	5.7 (3.8)
Tumor size benign, mean (SD), cm	4.4 (2.1)

**Table 2:** Logistic regression analysis and univariate models. Statistical significance level * 95%, ** 99%.

Univariate Logistic Regression Analysis			
Variable Name	*n* (%)	OR (95% CI)	*p*
Tumor size	201	1.12 (1.03–1.42)	0.032 *
≤3 cm	40 (19.9)	1—Reference	NA
3–4 cm	47 (23.4)	1.25 (0.48–3.23)	0.642
4–5 cm	36 (17.9)	1.29 (0.47–3.62)	0.629
>5 cm	78 (38.8)	2.61 (1.03–6.71)	0.043 *
Age	307	1.04 (1.02–1.06)	<0.001 **
<50 years	69 (22.5)	0.36 (0.18–0.72)	0.004 **
50–60 years	77 (25.1)	1.13 (0.55–2.32)	0.74
60–70 years	87 (28.3)	1.51 (0.74–3.16)	0.258
≥70 years	74 (24.1)	1—Reference	NA
Male	186 (60.6)	1.71 (1.05–2.81)	0.031 *
Right side	146 (47.6)	0.89 (0.55–1.45)	0.653
Year	307	1.07 (1.01–1.14)	0.022 *

**Table 3:** Logistic regression analysis and multivariate models. Statistical significance level * 95%.

Multivariate Logistic Regression Analysis				
Variable Name	Continuous		Categorized	
	OR (95% CI)	*p*	OR (95% CI)	*p*
Tumor size	1.22 (1.05–1.47)	0.023 *	NA	NA
≤3 cm	NA	NA	1–Reference	NA
3–4 cm	NA	NA	1.27 (0.48–3.40)	0.628
4–5 cm	NA	NA	1.48 (0.52–4.40)	0.465
>5 cm	NA	NA	3.21 (1.22–8.68)	0.019 *
Age	1.03 (1.00–1.06)	0.043 *	NA	NA
<50 years	NA	NA	0.46 (0.16–1.26)	0.131
50–60 years	NA	NA	0.72 (0.27–1.87)	0.499
60–70 years	NA	NA	1.64 (0.58–4.83)	0.354
≥70 years	NA	NA	1–Reference	NA
Male	1.72 (0.85–3.49)	0.133	1.78 (0.86–3.69)	0.120
Right side	0.76 (0.38–1.52)	0.438	0.83 (0.40–1.69)	0.604
Year	0.97 (0.88–1.07)	0.596	0.98 (0.89–1.08)	0.693

**Table 4:** Malignancy risk modeling for patients exhibiting median, lower and upper quartile characteristics.

Sex	Age	Tumor Dimension (cm)	Kidney	Malignancy Risk (%)	Risk Ratio
Female	51.75	4.6	Right	63.0	0.91
Female	61.2	4.6	Right	69.1	1.00
Female	69.75	4.6	Right	74.1	1.07
Female	61.2	3.5	Right	64.2	0.93
Female	61.2	6	Right	74.7	1.08
Male	51.75	4.6	Right	74.5	1.08
Male	61.2	4.6	Right	79.3	1.15
Male	69.75	4.6	Right	83.1	1.20
Male	61.2	3.5	Right	75.5	1.09
Male	61.2	6	Right	83.5	1.21

## Data Availability

The datasets used and/or analyzed during the current study are available from the corresponding author on reasonable request.
